# Chemical constituents and phytochemical properties of floral maize pollen

**DOI:** 10.1371/journal.pone.0247327

**Published:** 2021-02-24

**Authors:** Japar Sidik Bujang, Muta Harah Zakaria, Shiamala Devi Ramaiya

**Affiliations:** 1 Department of Biology, Faculty of Science, Universiti Putra Malaysia, Serdang, Selangor Darul Ehsan, Malaysia; 2 Department of Aquaculture, Faculty of Agriculture, Universiti Putra Malaysia, Serdang, Selangor Darul Ehsan, Malaysia; 3 International Institute of Aquaculture and Aquatic Sciences (I-AQUAS), Universiti Putra Malaysia (UPM), Port Dickson, Negeri Sembilan, Malaysia; 4 Department of Crop Science, Faculty of Agricultural Science and Forestry, Universiti Putra Malaysia Bintulu Sarawak Campus, Bintulu, Sarawak, Malaysia; Institute for Biological Research, SERBIA

## Abstract

Currently, bee-gathered pollen (bee pollen) is commonly used worldwide as a dietary supplement and is recognized for its curative properties. Floral pollen is also important but is less recognized due to a lack of investigation. This study aims to determine the morphological characteristics and nutritional and phytochemical properties of floral maize pollen. Fresh pollen grains harvested from a farm of maize plants are yellow in colour and spheroid in shape. They change to amber and indented prismatic solid shapes when dehydrated. The main composition of floral maize pollen is carbohydrates (44.30±3.73%), followed by moisture (23.38±5.73%), crude proteins (17.16±3.13%), crude fibres (9.56±0.92%), and ash (4.98±0.11%), while the lowest content is observed for crude fats (0.62±0.06%). The predominant mineral is potassium (768.50±11.40 mg 100 g^-1^), followed by sodium (695.10±9.70 mg 100 g^-1^), calcium (147.20±12.60 mg 100 g^-1^), and magnesium (97.30±2.9 mg 100 g^-1^). The microelements (with average values) consist of iron (49.50±3.30 mg 100 g^-1^) and zinc (30.00±3.70 mg 100 g^-1^). Excellent phytochemical properties add value to floral maize pollen. Maize pollen contains a high total phenolic content (TPC) and total flavonoid content (TFC) of 783.02 mg GAE 100 g^-1^ and 1706.83 mg QE 100 g^-1^, respectively, and possesses strong antioxidant activity of 10.54 mg mL^-1^. Maize floral pollen and derived products can serve as future food resources for human consumption and as a source of functional and bioactive compounds in nutraceutical and pharmaceutical industries.

## Introduction

Maize or corn (*Zea mays* L.) is a plant belonging to the Poaceae family. It is a monoecious and annual plant grown widely all over the world. There are different types of maize, e.g., feed corn (*Zea mays* var. *indenata*), flint corn (*Zea mays* var *indurata*) and sweetcorn (*Zea mays* var. *saccharata*). All parts of maize plants are useful, such as for food and feed for humans and livestock, respectively. Maize cobs provide a soft-grit abrasive and furfural. Extracted oil, bran, and starch come from the plant kernel [1]. The silk of maize is used for animal feed and silage. Maize husks are filling materials for dolls, whereas paper and wallboard come from the stalk of maize plants [2].

Male gametophytes of plant seeds produce pollen grains [3]. Pollen grains are living organisms, and both the environment and genotype influence their behaviour and survival. Pollen grains have various shapes, sizes, and surfaces. They possess nutritionally essential substances, such as carbohydrates, proteins, amino acids, lipids, and mineral substances [4]. Significant amounts of phytochemicals, including carotenoids, steroids, terpenes, and flavonoids, are present in floral maize pollen [3–6]. Pollen is used in pollination and as a food for insects [7]. In addition, pollen has gained attention for its therapeutic properties, such as its antibacterial [8, 9], anticariogenic [10] and immunomodulatory effects [11]. For centuries, apicultural products have been used in phytotherapy and diet due to their positive health implications [4, 6, 12–15]. Bee-gathered pollen (bee pollen) is an apicultural product of great commercial interest due to its high nutritional value and physiological properties, representing an important energy and protein source for human nutrition. Considering the positive effects of floral pollen nutrients and phytometabolites on human and animal health, floral pollen can serve as a future food resource and a source for product derivation [16].

According to statistics on maize production by the United States Department of Agriculture (USDA), the largest producer of the maize crop is the United States of America, with 347,782 tons in 2019, while Malaysia produced 58 tons of maize crops. Floral maize pollen was selected in this research due to its ample amount produced during anthesis. Therefore, instead of wasting these products, this research aims to investigate the utilization of useful maize pollen products as foods or dietary supplements for human health. For the above purpose, we assessed the nutritive properties, phenolics, flavonoid contents, and antioxidant activities of floral maize pollen.

## Materials and methods

### Sample collection and storage

*Zea mays* plant variety sweet corn D56 was planted on Plot 13, Shared Farm 2, Universiti Putra Malaysia Bintulu Sarawak Campus (N 03° 12.42’ and E 113° 4.95’), Sarawak. Fresh floral maize pollen was collected during anthesis from 5 plants randomly selected from the 20 planted maize plants. Pollens were collected into separate Ziplock bags by gently tapping the main stems of the maize plants. The 5 Ziplock bags containing floral maize pollen were brought immediately to the laboratory. The floral maize pollen was cleaned, sifted through a sieve, and placed into an airtight container. The pollen, either fresh or stored at -20°C, was subsequently used for the various analyses described below.

### Pollen morphological observation

The detailed morphological structures of the anther, fresh, and dehydrated pollens were examined under a 3D microscope (Keyence VHX-600), and their sizes were recorded.

### Proximate analysis of the floral maize pollens

Proximate analysis of the moisture, ash content, crude proteins, crude fats, and crude fibre composition of floral maize pollen was determined using the standard methods of the Association of Official Analytical Chemists [17]. The moisture content of the pollen samples was determined by drying each sample until a constant weight was obtained. The ash value was determined by incinerating air-dried samples in a muffle furnace at 550°C for 5–6 hours (method 930.05). The percentage of crude protein content was determined by multiplying the percentage of nitrogen content obtained from the samples using Kjeltec Auto Distillation 2200 Foss by a factor of 6.25 (method 955.04). The crude lipid from the sample was extracted using petroleum ether as the solvent. Crude lipids were determined using the 2055 Soxtec Avanti Manual System, Sweden (method 920.39). The crude fibre was estimated by acid-base digestion based on method 993.19. The total carbohydrate content in the samples was calculated by using the formula [100 − (%Crude Protein + %Crude Fat + %Crude Fibre + %Ash)] [18].

### Mineral content analysis of the floral maize pollen

The ash obtained from the ash content determination was used to extract the minerals using the dry-ashing method following the AOAC [17]. The mineral elements calcium (Ca), potassium (K), sodium (Na), magnesium (Mg), iron (Fe), zinc (Zn) and copper (Cu) concentrations were determined by atomic absorbance spectrophotometry (AA800 Perkin Elmer, Germany) based on method 975.03 [17].

### Ethanolic pollen extraction

Two (2.0) grams of crushed maize pollen were extracted with 15 mL of a solution containing 70% ethanol:30% water (v/v). The samples were vortexed and placed in an ultrasonic bath at room temperature for 30 min. The extracts were centrifuged at 1640 x ɡ for 15 min at room temperature and filtered through Whatman No. 2 filter paper. The filtered extracts were used for the determination of total phenolic content (TPC), total flavonoid content (TFC), and antioxidant activity (AA).

### Determination of total phenolic content (TPC)

The total phenolic content was determined by the Folin-Ciocalteu method as used by Ramaiya et al. [19]. The absorbance readings were recorded at 740 nm on a 1100 Series Spectrophotometer. Quantification of TPC was performed using a calibration curve prepared with a gallic acid standard ranging from 0 to 500 mg 100 g^-1^, and the results were expressed as mg gallic acid equivalents (GAE) per 100 g pollen extracts. The obtained linear gallic acid standard curve was y = 0.003x+0.079 with R^2^ = 0.990.

### Determination of total flavonoid content (TFC)

The total flavonoid content was determined by the aluminium chloride colorimetric assay following the method of Meda et al. [20]. The absorbance readings were taken against a blank at 510 nm on a 1100 Series Spectrophotometer. The total flavonoid contents were expressed as mg of quercetin equivalents (QE) per 100 g of pollen extracts. The obtained linear quercetin standard curve was y = 0.000x+0.006 with R^2^ = 0.992.

### Determination of antioxidant activity (AA)

The antioxidant activity was determined using the *2*,*2-diphenyl-1-picrylhydrazyl* (DPPH) method based on quantifying the free radical scavenging activity of the extracts described by Ramaiya et al. [21]. The absorbance was recorded against a blank at 517 nm on a spectrophotometer. Inhibition of free radicals by DPPH as a percentage was calculated using the following formula:

$$Inhibition\;of\;DPPH(\%)=\frac{Ab-Aa}{Ab}\;X100,$$ (1)

where Ab is the absorption of the blank sample and Aa is the absorption of the pollen extract. The concentration of each sample required to scavenge 50% DPPH (EC_50_) expressed as mg mL^-1^ was determined by linear regression of inhibition percentage against juice concentration. The DPPH radical scavenging activity was expressed as mg mL^-1^. A lower EC_50_ value indicates higher antioxidant activity.

### Statistical analysis

Means, standard errors, and ranges for the proximate compositions, mineral content, and antioxidant activity were computed for five sample determinations. The EC_50_ values for AA were calculated by linear regression analysis. The nutrient contents of *Z*. *mays* pollen and other floral pollens from various studies, i.e., saffron (*Crocus sativus*), date palm (*Phoenix dactylifera*), sunflower (*Helianthus annuus*), alfalfa (*Medicago sativa*), rape (*Brassica napus*), olive (*Olea europaea*), rose (*Rosa laxa*), oil palm (*Elaeis guineensis*), and bee-gathered pollen, i.e., bee pollen (*Apis mellifera*), bee pollen (*Melipona interrupta*), bee pollen (*Melipona subnitida*), bee pollen (monofloral) and bee pollen (polyfloral), were ordinated with principal component analysis (PCA). The ordination to obtain the relationship between variables and pollen was based on the Pearson method using XLSTAT software version 2013.5. Clustering was conducted using hierarchical cluster analysis to specify the distance or similarity measure used in clustering with Ward’s method. The analyses were performed using XLSTAT 2013.5 for Windows.

## Results and discussion

### Morphological characteristics of floral maize pollen

**Fig 1:**
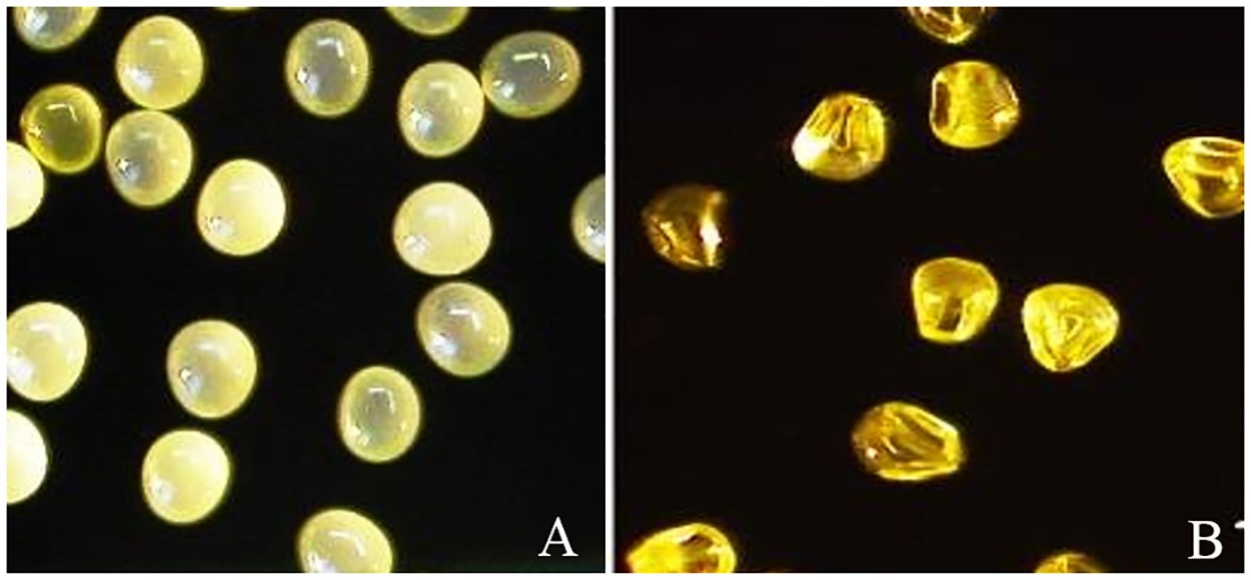
Morphology of floral maize pollen under KeyenceVHX 600 Digital Microscope (a) fresh maize pollen (magnification 200x) and (b) shrunk maize pollen after dehydration (magnification 200x).

The release of pollen grains can start from sunrise until noon depending on the plant’s temperature, humidity and genetic constitution [22]. The pollen was harvested as soon as anthesis occurred during the 6^th^ and 7^th^ weeks after planting at approximately 9.30 am to 12.30 pm. Studies by Kaefer et al. [22] stated that the best results from viable pollen grains were obtained in the morning and that ambient temperature and relative humidity were the main factors influencing pollen viability rather than the time of day. As the anthers of the *Z*. *mays* plant dehisce, they split apart to allow pollen grain to fall into the open air. Pollen grains are viable for only a few minutes after shading until they desiccate. Ferreira et al. [23] reported that maize pollen grain does not have much strength and can lose viability within a range of one to four hours after being released into the atmosphere. A tassel typically sheds pollens for approximately five days. Pollen shed in a field can last up to two weeks. There is a slight difference in the width and length of fresh and shrunken pollen. The size of fresh pollen is 10.23±0.60 μm (range from 9.36 to 11.15 μm) x 9.17±0.59 μm (range from 8.28 to 10.09 μm), while the size of shrunken pollen is 9.87±0.38 μm (range from 9.52 to 10.81 μm) x 8.11±0.77 μm (range from 6.74 to 9.18 μm). The fresh pollen shape changed from a prolate spheroid to an indented, prismatic solid (Fig 1a) and changed colour from yellow to amber when dehydrated. The pollen is yellow due to its main flavonoid, quercetin [24]. High temperature and low humidity of the environment shrinks maize pollen. Maize pollen is very sensitive to high temperature and desiccation. The shrunk pollen resembles the seeds of the maize itself (Fig 1b). According to Aylor [25], floral maize pollen is sensitive to dehydration and rehydration. The deterioration of pollen during storage and drying involves many physical and chemical changes, including changes in odour, taste, colour, and shape, disrupted intracellular integrity, decreased enzyme activities, lipid peroxidation and phenolic oxidation.

### Proximate composition of floral maize pollen

Table 1 shows the proximate composition of floral maize pollen and other floral pollens from various authors’ studies. Categorically, the proximate composition of maize pollen is represented as carbohydrates > moisture > crude proteins > ash > crude fibres > crude fats.

**Table 1:** Proximate composition of floral maize pollen in the present study and comparison with pollen from previous studies.

Variables	Proximate composition (%)						Reference
	Moisture	Ash	Crude fibres	Crude fats	Crude proteins	Carbohydrates	
Maize (*Zea mays*)	23.38±5.73 (15.10–29.80)	4.98±0.11 (4.83–5.15)	9.56±0.92 (8.60–11.00)	0.62±0.06 (0.55–0.70)	17.16±3.13 (13.13–20.14)	44.30±3.73 (41.51–50.51)	Present study
Maize (*Zea mays*)	34.84	2.22	5.34	1.32	16.47	39.81	[26]
Saffron (*Crocus sativus*)	12.50	9.50	7.40	5.80	23.60	20.00	[27]
Date palm (*Phoenix dactylifera*)	28.80	4.57	1.37	20.74	31.11	13.41	[28]
Sunflower (*Helianthus annuus*)	9.19	2.01	1.70	4.45	14.71	67.95	[29]
Alfalfa (*Medicago sativa*)	9.84	3.74	0.78	2.89	19.45	63.3	[29]
Rape (*Brassica napus*)	9.73	3.32	0.46	3.92	18.14	64.43	[29]
Rose (*Rosa laxa-*Alar)	52.62	1.06	10.24	1.35	8.25	26.48	[29]
Rose (*Rosa laxa-*Tianshan)	53.25	1.11	8.13	2.03	6.62	28.86	[30]
Oil palm (*Elaeis guineensis*)	10.84	3.96	0.91	0.92	21.86	61.51	[31]
Olive	28.50	6.52	2.53	30.63	40.01	20.31	[32]
Palm	29.00	6.20	2.30	31.50	39.80	20.20	[32]
Bee pollen (*Apis mellifera*)	4.20	2.90	3.40	4.90	20.50	64.10	[33]
Bee pollen (*Melipona interrupta*)	37.12	2.74	13.65	6.47	24.00	44.27	[34]

Data are displayed with mean values ± S.D. and the ranges are in parentheses (n = 5).

**Fig 2:**
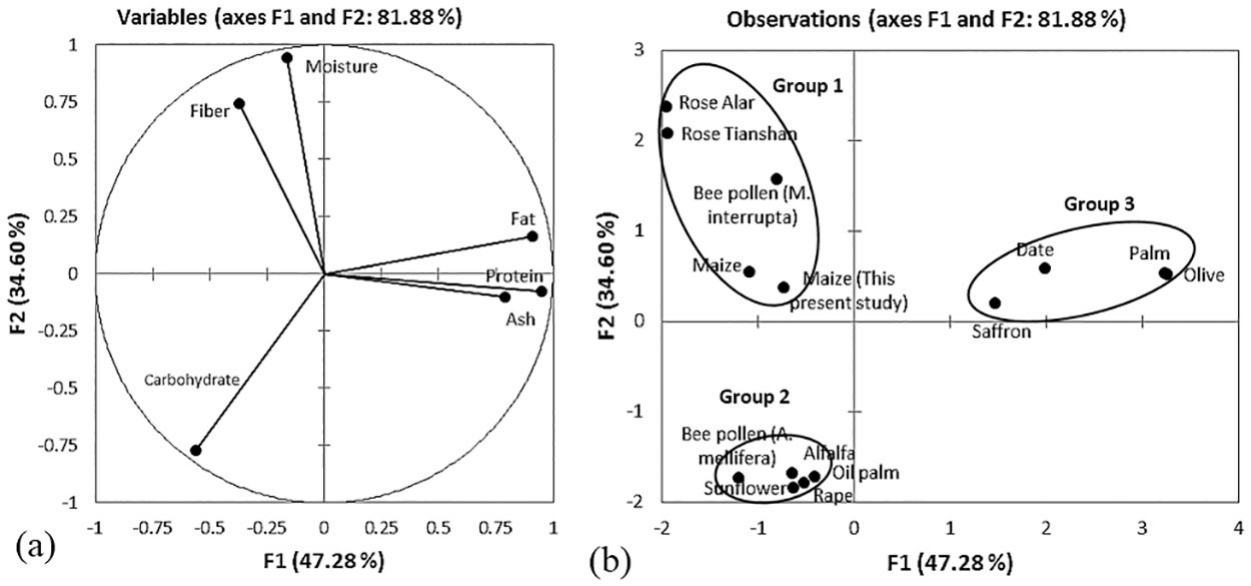
(a) Plot of the variables tested for proximate content, where the percentage in parentheses represents the variation of each component, and (b) positions of the PC scores of the 13 pollen types according to F1 and F2 for proximate content.

Based on PCA, the maize pollen parameters were comparable with other floral pollens studied by various researchers, i.e., saffron, date palm, sunflower, alfalfa, rape, olive, palm, rose, oil palm and bee pollen. The first two PCs accounted for 81.88% of the total variance. PC1 explained a higher percentage of the total variance, 47.28%, than PC2 (34.60%). Fig 2a shows the variables for proximate analysis, consisting of crude fats, crude proteins, and ash, which were highly connected to the positive side of PC1. In contrast, moisture, crude fibres, and carbohydrates were connected to the negative side of PC1.

Fig 2b shows fourteen various pollens, including the maize pollen in the present study, clustered into three main groups. The first group consists of the studied maize pollen, previously studied maize pollen, *Rosa laxa*-Alar, *Rosa laxa*-Tianshan pollen and pollen collected by bees (*Meliponini interrupta*). They are clustered together due to their similar higher moisture content and fibre composition.

Moisture content is vital to ensure the stability and quality of pollen. Fresh and dry pollen loads have different water contents, ranging from 20–30% in the original form and 4–10% if dried, affecting organoleptic and “shelf lifetime” properties [35]. The moisture content of floral maize pollen was lower (23.38±5.73%) than the reported value (34.84%) [26]. In addition, the fibre content of floral maize pollen was higher (9.56±0.92%) than that reported in a previous study (5.34%) by Andronescu [26]. Comparatively higher fibre composition (13.65%) was recorded in pollens gathered by stingless bees, *Meliponini interrupta* [34] and the floral pollen of roses [30], ranging from 8.13 to10.24%.

A second group is pollen from bee pollen (*A*. *mellifera*), together with the floral pollen of alfalfa, oil palm, rape, and sunflower pollen. This group possesses a higher carbohydrate content ranging from 61.51 to 67.95%. The carbohydrate content of the examined maize pollen was 44.30±3.73%, comparable to pollens gathered by *Meliponini interrupta* bees [34]. The higher amounts of carbohydrates and crude fibres indicate that maize pollen could serve as a source of energy and food fibre and make it a potential food ingredient. The third group consists of pollen from saffron, date palm, palm, and olive due to their similar ash, crude fat, and protein contents. The ash content of saffron pollen is approximately two times higher (9.50%) than that of maize pollen (4.98±0.11%). The obtained value for maize pollen was nearly two times higher than the value of 2.22% reported by Andronescu [26]. A higher ash amount indicates that the pollen contains high concentrations of various minerals. This is in agreement with the finding of Sani et al. [27], who noted that saffron pollen is a rich source of mineral elements. Comparatively, the ash content of floral maize pollen was two times higher than that of bee pollen at 2.74% and 2.90% [33–34]. The pollen of olive, palm, and date palm possessed a relatively higher fat content, ranging from 20.74–31.50%, compared to that of the examined and previously investigated floral maize pollen, with the lowest fat contents, 0.62±0.06 and 1.32%, respectively. Higher protein content was from the date palm, palm, and olive pollens with 31.11%, 40.01%, and 39.80%, respectively. The protein content in floral maize was two times lower (17.16±3.13%) than that in members of this group. Variation of the nutrient content of floral pollen by species could be due to environmental conditions during maturation and the age and vigour of the plants [32], soil type, beekeeping management, climatic and preservation conditions [36, 37] and the botanical origin and its genetic variability [38].

**Fig 3:**
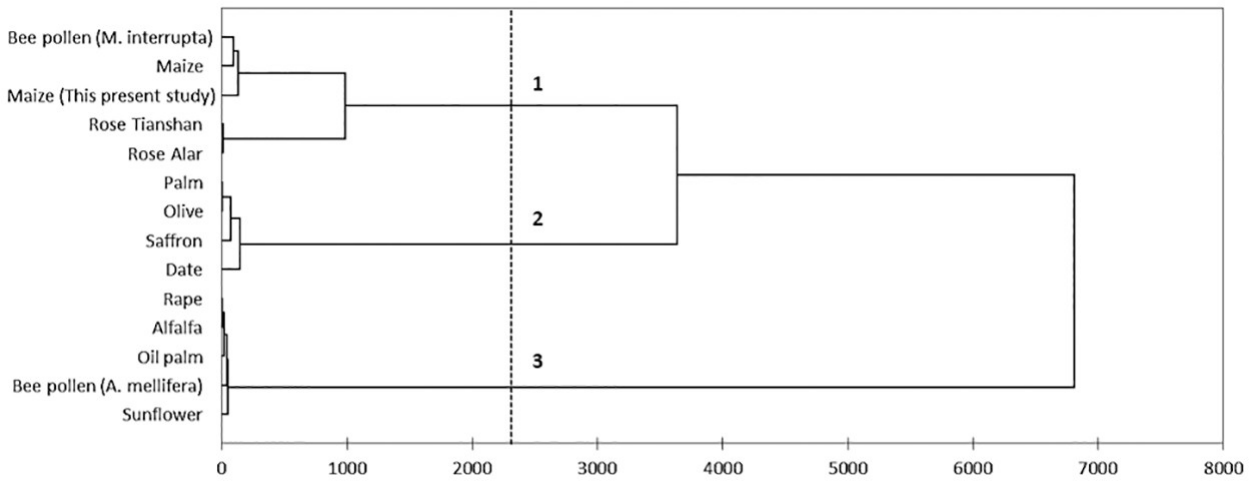
Cluster dendrogram of Ward’s method based on proximate compositions of various pollens.

Fig 3 shows the assessment of the proximate composition of produced clustered pollens according to their composition similarities, as reflected in the hierarchical cluster analysis dendrogram. The results were similar to clustering using PCA in Fig 2. The obtained dendrogram separates the different pollen studied into three distinct groups with no overlaps. Group 1 consisted of the studied maize pollen, previously reported maize pollen, *Rosa laxa*-Alar, *Rosa laxa*-Tianshan pollen, and pollen from stingless bee species (*M*. *interrupta*). Group 2 comprised bee pollen (*A*. *mellifera*), alfalfa, oil palm, rape, and sunflower pollen. Pollens of saffron, date palm, palm, and olive clustered in Group 3. The variance composition for clustering was 14.94% within classes and 85.06% between classes.

### Mineral content of floral maize pollen

**Fig 4:**
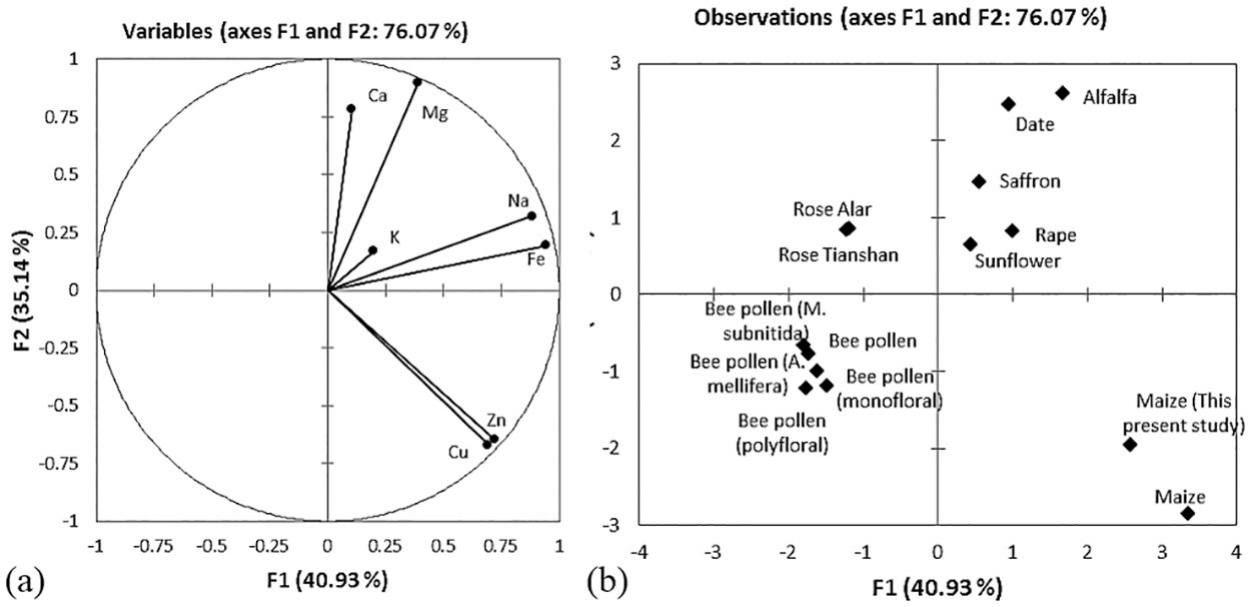
(a) Plot of the variables tested for mineral content, where the percentage in parentheses represents the variation of each component, and (b) positions of the PC scores of the 14 pollen types according to the F1 and F2 for mineral content.

Minerals are important in determining the nutritional value of pollen. The ash content of maize pollen is an indication that pollen provides a considerable amount of minerals that are essential for the body. Table 2 shows the macronutrient and micronutrient contents of maize pollen. Maize pollen contains K, Ca, Mg, Na, Fe, Cu, and Zn in varying concentrations. The trend of mineral content in maize pollen studied was categorically as follows: K > Na > Ca > Mg > Fe > Zn > Cu. Fig 4 illustrates the biplot ordinated with PCA, which shows the mineral amounts compared with other floral pollens, i.e., saffron, date palm, sunflower, alfalfa, rape, rose, and various sources of bee pollen. The PCA indicated that the first two PCs for pollen accounted for 76.07% of the total variance. PC1 explained a higher percentage of the total variance (40.93%) than PC2 (35.14%), as shown in Fig 4a.

**Table 2:** Mineral content of floral maize pollen.

Variables	Mineral content (mg 100 g^-1^)							Reference
	K	Mg	Na	Ca	Fe	Cu	Zn	
Maize (*Z*. *mays*)	768.5±8.4 (762.0–801.0)	97.3±2.9 (87.5–103.0)	695.1±9.7 (654.6–723.0)	147.2±9.6 (122.0–175.0)	49.5±3.3 (38.5–58.0)	15.7±0.6 (14.5–17.3)	30.0±3.7 (23.3–38.5)	Present study
Maize (*Z*. *mays*)	1059.0	115.8	593.0	92.0	48.0	20.0	19.0	[39]
Saffron (*C*. *sativus*)	540.0	410.0	560.0	230.0	43.4	0.4	1.2	[40]
Date palm (*P*. *dactylifera*)	640.3	468.0	658.6	562.9	33.8	0.4	4.1	[29]
Sunflower (*H*. *annuus*)	623.3	270.4	634.5	208.6	34.3	0.7	3.4	[29]
Alfalfa (*M*. *sativa*)	721.4	455.7	731.1	575.2	53.3	0.6	4.4	[29]
Rape (*B*. *napus*)	825.9	388.7	835.0	524.6	36.1	0.6	3.5	[29]
Rose (*R*. *laxa-*Alar)	1490.0	230.0	11.0	377.0	5.2	0.4	2.2	[30]
Rose (*R*. *laxa-*Tianshan)	1313.0	257.0	17.0	355.0	4.8	0.4	2.2	[30]
Bee pollen (*A*. *mellifera*)	511.6	75.4	20.2	103.1	7.9	1.1	5.0	[33]
Bee pollen (monofloral)	351.2	87.5	3.4	123.6	9.4	0.7	4.0	[41]
Bee pollen (polyfloral)	371.1	65.7	2.1	97.3	4.8	0.6	4.3	[41]
Bee pollen (*M*. *subnitida*)	591.8	97.5	nd	186.4	1.6	0.1	3.6	[41]
Bee pollen	408.6	70.2	11.1	216.6	4.2	0.9	3.7	[42]

Data are displayed with mean values ± S.D. and the ranges are in parentheses (n = 5).

The biplot generated four main groups based on their mineral amounts in Fig 4b. The first group consisted of pollen of five species: alfalfa, date palm, saffron, rape, and sunflower. This group includes pollen that has high levels of all macronutrients, i.e., potassium, calcium, magnesium, and sodium, and micronutrients as well as iron. The K contents of members of this group were saffron (540.0 mg 100 g^-1^), date palm (640.3 mg 100 g^-1^), sunflower (623.3 mg 100 g^-1^) and alfalfa (721.4 mg 100 g^-1^). The value for the floral maize pollen (768.5±11.4 mg 100 g^-1^) fell within this range. However, the K content of previously reported floral maize pollen (1059.0 mg 100 g^-1^) was higher than the present value [42]. An adequate amount of K intake helps lower urinary calcium excretion and manage hypercalciuria and kidney stones in addition to decreasing the risk of osteoporosis [43]. Calcium is essential in bone formation and strength. The Ca content of the floral maize pollen examined was 147.2±12.6 mg 100 g^-1^, and the previously reported value was 92.0 mg 100 g^-1^. Comparatively, these values were approximately twice as low as those of saffron pollen (230 mg 100 g^-1^) [40] and four times lower than those of date palm (562.9 mg 100 g^-1^) [29]. Bello et al. [44] reported that magnesium is important in neurochemical transmission and muscular excitability.

The magnesium content of saffron, date palm, and alfalfa pollen was four times lower than that of the examined maize pollen, which was 97.3±2.9 mg 100 g^-1^. The magnesium content of bee pollen, i.e., pollen obtained by the bee of *M*. *subnitida* (97.5 mg 100 g^-1^), was similar to that of maize pollen. The sodium content of floral maize pollen (695.1±9.7 mg 100 g^-1^) was consistent with members of this group, i.e., saffron, date palm, sunflower, and alfalfa ranged from 560.0–731.1 mg 100 g^-1^. Similarly, the iron content of the presently examined maize pollen and previously reported maize pollen ranged from 48–49.5 mg 100 g^-1^; these values were within the range for members of this group, i.e., date palm pollen (33.8 mg 100 g^-1^), saffron pollen (43.4 mg 100 g^-1^) and alfalfa pollen (53.3 mg 100 g^-1^). Floral maize pollen is a rich source of nonheme or plant-based iron. Iron is very important in the formation of red blood cells [44].

The second group consisted of the examined floral maize pollen and previously reported maize pollen, which correlated with the micronutrients copper and zinc. The floral maize pollen exhibited a higher copper content of 15.7±0.6 mg 100 g^-1^, similar to the previously recorded value in maize of 20.0 mg 100 g^-1^, while other floral pollens have a lower content, i.e., sunflower (0.7 mg 100 g^-1^), bee pollen collected by *A*. *mellifera* (1.1 mg 100 g^-1^) and *R*. *laxa* (0.4 mg 100 g^-1^). Comparatively, the Zn content of maize pollen (30.0±3.7 mg 100 g^-1^) was higher than that of saffron (1.2 mg 100 g^-1^), date palm (4.1 mg 100 g^-1^) and sunflower (3.4 mg 100 g^-1^) floral pollens. All the bee pollens were clustered in the third group with the lowest mineral concentration compared with the other pollens. The fourth group comprised rose pollen, *Rosa laxa*-Alar and *Rosa laxa*-Tianshan, with higher K contents (range from 1313 to 1490 mg 100 g^-1^) and lower Na contents (range from 11 to 17 mg 100 g^-1^).

**Fig 5:**
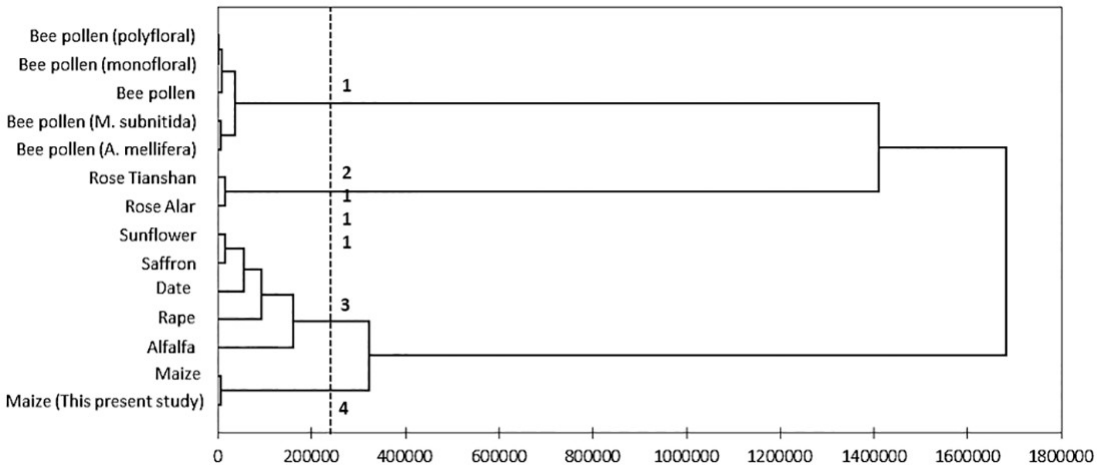
Cluster dendrogram of Ward’s method based on the mineral content of various pollens.

Fig 5 shows the hierarchical cluster analysis dendrogram of the mineral content of pollen according to the macronutrient and micronutrient concentration similarities. The results followed the clustering using PCA in Fig 4. The obtained dendrogram separates all the pollen studied into four distinct groups with no overlaps. Group 1 consisted of pollen of alfalfa, date palm, saffron, rape, and sunflower. The examined maize pollen and previously reported maize pollen stood independently in a separate group and were clustered in Group 2. All the bee pollens were in Group 3, and Group 4 comprised rose pollens: *Rosa laxa*-Alar and *Rosa laxa*- Tianshan. The variance composition for clustering was 5.93% within classes and 94.07% between classes.

### Total phenolic content (TPC), total flavonoid content (TFC) and antioxidant activity (AA) of floral maize pollen

Various experiments have been conducted on the potential effects of natural antioxidants from floral pollen for edible and industrial uses. The phenolic content present in pollen, e.g., olive, palm, pine, and bee pollen, has been discovered, which offers exciting nutritional and therapeutic possibilities [33, 45–47]. Concerning maize pollen, the total phenolic content (TPC) was 783.02±37.01 mg GAE 100 g^-1^, ranging from 741.94 to 843.38 mg 100 g^-1^, and the total flavonoid content (TFC) ranging from 1678.03 to 1770.10 mg QE 100 g^-1^, with a mean value of 1706.83±39.46 mg QE 100 g^-1^ (Table 3). The phenolic content results are within those of Zilic et al. [48], which are 777.93 mg GAE 100 g^-1^ (yellow maize) and 993.30 mg GAE 100 g^-1^ (sweet maize). The flavonoid content obtained from the present study was higher than that reported by Zilic et al. [48], which was 892.83 mg QE 100 g^-1^ (yellow maize) and 1500.10 mg QE 100 g^-1^ (sweet maize). In comparison, the TPC and TFC of maize pollen were two times (353 mg GAE 100 g^-1^) and six times (270 mg QE 100 g^-1^) higher, respectively, than those of fresh maize kernels [49].

**Table 3:** Total phenolic content (TPC), total flavonoid content (TFC), and antioxidant activity (AA) of floral maize pollen.

Variables	Values
Total phenolic content (TPC) (mg/100 g)	783.02±37.01 (741.94–843.38)
Total flavonoid content (TFC) (mg/100 g)	1706.83±39.46 (1678.03–1770.10)
Total antioxidant activity (TAA) (mg/mL)	10.54±1.18 (9.39–11.81)

Data are displayed with mean values ± S.D. and the ranges are in parentheses (n = 5).

According to the present results, maize pollen is richer in TFC than in TPC. Goss [18] revealed that maize pollen is yellow because of the presence of the flavonoid pigment quercetin and its derivatives. The flavonoid pattern of maize pollen is characterized by the accumulation of the most dominant flavonols, quercetin and trace levels of isorhamnetin diglycosides and rutin [48]. According to Lundgren and Wiedenmann [50] and Zilic et al. [48], the quercetin values in maize pollen were 324.16 μg g^-1^ and 81.61 to 466.82 μg g^-1^, respectively. The presence of quercetin identifies antioxidant activities in plant materials. Quercetin is a beneficial protective agent that reduces oxidative damage to important biomolecules, including lipoprotein and DNA (deoxyribonucleic acid), from reactive oxygen species [51].

Similar to the total phenolic and flavonoid contents, the strongest antioxidant activity was in the maize pollen extract. The lower the EC_50_ values, the higher the antioxidant activity in maize pollen extract. The total antioxidant activity of the maize pollen extract was 10.54±1.18 mg mL^-1^, ranging from 9.39 to 11.81 mg mL^-1^. The present TAA values were lower than those of previously investigated floral maize pollen and bee pollen (*A*. *mellifera*) extracted using methanol, at 0.36 mg mL^-1^ and 0.42 mg mL^-1^, respectively [52]. The lower antioxidant activity obtained in the present study was attributed to the extraction method, which caused the loss of natural antioxidant compounds, as reported by Nicoli et al. [53]. Using the ABTS assay, Zilic et al. [48] studied seven floral pollen samples from different maize genotypes, with sweet maize pollen showing a TAA value of 104.38 mmol Trolox kg^-1^. This level was higher (24%) than that found in yellow maize pollen, 79.94 mmol Trolox kg^-1^.

In general, a higher total phenolic content may lead to a higher value of antioxidant activities. Several authors have mentioned this relationship in various studies [48, 49]. Table 4 shows the significant positive and strong correlation obtained between TPC and TAA (*r* = 0.997). This suggests that TPC is the main contributor to the antioxidant activity in maize pollen. Javanmardi et al. [54] indicated that the antioxidant activities are contributed by the total phenolic content from flavonoids and other antioxidant secondary metabolites, such as volatile oils, carotenoids, and vitamins. Studies have indicated that these phytochemicals have high free-radical scavenging activity, which helps reduce the risk of chronic diseases, such as cardiovascular disease and cancer [55]. Currently, bee pollen represents the richest and most complete natural food supplying high levels of carbohydrates (13–55%) and proteins (10–40%), particularly free amino acids, enzymes, cofactors, and lipids (1–13%), including fatty acids and sterols and vitamins [35, 36]. Moreover, pollen gathered by bees constitutes a natural source of antioxidants, phenolic acids and flavonoids responsible for biological activities that can regulate intestinal functions and have beneficial effects on the cardiovascular system [4, 56]. It also helps prevent prostate problems, arteriosclerosis, gastroenteritis, and respiratory diseases [12, 13].

**Table 4:** Correlation matrix for TPC, TFC and TAA.

Variables	TPC	TFC	AA
TPC	1		
TFC	0.993	1	
AA	**0.997**	0.981	1

Values in bold are significant at *p*< 0.05.

In particular, the bee pollen phenolic profile consists of flavanol, glycosides and aglycones, and hydroxycinnamic acids that can be present in free forms or combined with other pollen components [57]. The effects of pollens on improving immune, cardiovascular and digestive systems and their therapeutic effects have been mainly related to the polyphenol content and chemical composition [35]. Additionally, maize pollen was observed to possess good lipid content [43], and fatty acids (FAs) are an important part of the lipid fraction in pollen and could serve as a type of bioactive compound. Maize pollen could be used as a good source of unsaturated fatty acids (UFAs); the samples showed a higher prevalence of unsaturated fatty acids than saturated fatty acids (SFAs) (UFA/SFA ratio > 1.6). Consumption of pollen could supply a significant quantity of ω-3 and ω-6 fatty acids in the human diet [58]. The good nutritional properties and phytochemical constituents of floral maize pollen strongly support its ethnobotanical perspective in traditional medicine to treat various infectious diseases. Floral maize pollen could produce benefits if used as a functional food ingredient and dietary supplement with therapeutic effects.

## Conclusions

The present study found that floral maize pollen possessed higher nutritional values and a beneficial combination of antioxidant compounds, mostly phenolics. There was a strong positive correlation between total phenolic compound content and antioxidant activity in floral maize pollen. Floral maize pollen can serve as a future food resource and derived product for human consumption and as a source of functional and bioactive compounds in nutraceutical and pharmaceutical industries, giving the plants value beyond their fruits. Future investigations should identify the active compounds that affect the free radical scavenging activities.

## Supporting information

S1 Data (XLSX)

## References

[1] Naves MMV, Castro MVLD, Mendonça ALD, Santos GG, Silva MS. Corn germ with pericarp in relation to whole corn: nutrient contents, food and protein efficiency, and protein digestibility-corrected amino acid score. Food Sci Tech. 2011;31(1):264–269. 10.1590/S0101-20612011000100040.

[2] Ranum P, Peña‐Rosas JP, Garcia‐Casal MN. Global maize production, utilization, and consumption. Annals of The New York Academy of Sci. 2014;1312(1):105–112. 10.1111/nyas.12396. 24650320

[3] de Arruda VAS, Pereira AAS, Freitas AS, Barth OM, Almeida-Muradian LB. Dried bee pollen: B complex vitamins, physicochemical and botanical composition. J Food Compos Anal. 2013;29:100–105. 10.1016/j.jfca.2012.11.004.

[4] Rzepecka-Stojko A, Stojko J, Kurek-Górecka A, Górecki M, Kabała-Dzik A, Kubina R, et al. Polyphenols from bee pollen: structure, absorption, metabolism and biological activity. Molecules 2015;20(12):21732–21749. 10.3390/molecules201219800 26690100 PMC6332396

[5] Mejías E, Montenegro G. The antioxidant activity of Chilean honey and bee pollen produced in the Llaima Volcano’s zones. J Food Qual. 2012;35(5):315–322. 10.1111/j.1745-4557.2012.00460.x.

[6] Alicic D, Subaric D, Jasic M, Pasalic H, Ackar D. Antioxidant properties of pollen. Hrana U Zdravlju I Bolesti. 2014;3(1):6–12.

[7] McQuate GT, Jones GD, Sylva CD. Assessment of corn pollen as a food source for two tephritid fruit fly species. Environ Entomol. 2003;32(1):141–150. 10.1603/0046-225X-32.1.141.

[8] Garcia M, Perez-Arquilue C, Juvan T, Juan MI, Herrera A. Note: pollen analysis and antibacterial activity of Spanich honeys. Food Sci Technol Int. 2001;7:155–158. 10.1177/108201320100700208.

[9] Gabriele M, Parri E, Antonio F, Sagona S, Pozzo L, Biondi C, et al. Phytochemical composition and antioxidant activity of Tuscan bee pollen of different botanic origins. Ital J Food Sci. 2015;27(2):248–259. 10.14674/1120-1770/ijfs.v191.

[10] Almas K, Mahmoud A, Dahlan AA. Comparative study of propolis and saline application on human dentin: A SEM study. Indian J Den Res, 2001;12:21–70. 11441797

[11] Gebara EC, Lima LA, Mayer M. Propolis antimicrobial activity against periodontopathic bacteria. Braz J Microbiol. 2002;33(4):365–369. 10.1590/S1517-83822002000400018.

[12] Kocot J, Kiełczykowska M, Luchowska-Kocot D, Kurzepa J, Musik I. Antioxidant potential of propolis, bee pollen, and royal jelly: Possible medical application. Oxidative medicine and cellular longevity 2018. 10.1155/2018/7074209. PMC5954854 29854089

[13] Ozcan MM, Aljuhaimi F, Babiker EE, Uslu N, Ceylan DA, Ghafoor K, et al. Determination of antioxidant activity, phenolic compound, mineral contents and fatty acid compositions of bee pollen grains collected from different locations. Journal of Apicultural Science 2019;63(1):69–79. 10.2478/jas-2019-0004.

[14] Barbieri D, Gabriele M, Summa M, Colosimo R, Leonardi D, Domenici V, et al. Antioxidant, nutraceutical properties, and fluorescence spectral profiles of bee pollen samples from different botanical origins. Antioxidants 2020;9(10):1001. 10.3390/antiox9101001. PMC7602780 33076547

[15] Yang Y, Liu M, Wang K, Yang Y, Su N, Huang W, et al. Chemical and cytological evaluation of honeybee pollen antioxidant ability. J Food Sci. 2020;85(3):824–833. 10.1111/1750-3841.15047 32078757

[16] Kostic AŽ, Milinčić DD, Barać MB, Ali Shariati M, Tešić ŽL, Pešić MB. The application of pollen as a functional food and feed ingredient-The present and perspectives. Biomolecules 2020;10(1):84. 10.3390/biom10010084 31948037 PMC7023195

[17] AOAC. Official Method of analysis. Washington, DC: Association of Official Agricultural Chemists; 2000.

[18] Winton AL, Winton KB. Techniques of Food Analysis. Jodhpur, Agrobios (India); 2006.

[19] Ramaiya SD, Bujang JS, Zakaria MH. Physicochemical, fatty acid and antioxidant properties of passion fruit (*Passiflora* species) seed oil. Pakistan J Nutr. 2019;18(5):421–429. 10.3923/pjn.2019.421.429.

[20] Meda A, Lamien CE, Romito M, Millogo J, Nacoulma OG. Determination of the total phenolic, flavonoid and proline contents in Burkina Fasan honey, as well as their radical scavenging activity. Food Chem. 2005;91(3):571–577. 10.1016/j.foodchem.2004.10.006.

[21] Ramaiya SD, Bujang JS, Zakaria MH, King WS, Shaffiq Sahrir MA. Sugars, ascorbic acid, total phenolic content and total antioxidant activity in passion fruit (*Passiflora*) cultivars. J Sci Food Agri. 2013;93(5):1198–1205. 10.1002/jsfa.5876. 23027609

[22] Kaefer KAC, Chiapetti R, Fogaça L, Muller AL, Calixto GB, Chaves EIDO. Viability of maize pollen grains in vitro collected at different times of the day. African Journal of Agricultural Research 2016;11(12):1040–1047. 10.5897/AJAR2015.10181.

[23] Ferreira CA, Voz Pinho EVR, Alvim PO, Andrade V, Silva TTA, Cardoso DL. Conservação e determinação da viabilidade de grão de pólen de milho. Rev. Bras. Milho e Sorgo 2007;6(2):159–173. 10.18512/1980-6477/rbms.v6n2p159-173.

[24] Freire KR, Lins A, Dórea MC, Santos FA, Camara CA, Silva T. Palynological origin, phenolic content, and antioxidant properties of honeybee-collected pollen from Bahia, Brazil. Molecules. 2012;17(2):1652–1664. 10.3390/molecules17021652 22314384 PMC6268123

[25] Aylor DE. Rate of dehydration of corn (*Zea mays* L.) pollen in the air. J Expl Bot. 2003;54:2307–2312. 10.1093/jxb/erg242 12909689

[26] Andronescu DI. The physiology of the pollen of Zea mays with special regard to vitality. [Thesis for Degree of Ph.D]. University of Illinois; 1915.

[27] Sani AM, Kakhki AH, Moradi E. Chemical composition and nutritional value of saffron’s pollen (*Crocus sativus* L.). Nutr Food Sci. 2013;43(5):490–495. 10.1108/NFS-04-2012-0040.

[28] Hassan HMM. Chemical composition and nutritional value of palm pollen grains. Global J Biotechnol Biochem. 2011;6(1):1–7.

[29] Taha EKA. Chemical composition and amounts of mineral elements in honeybee-collected pollen in relation to botanical origin. J Apic Sci. 2015;59(1): 75–81. 10.1515/jas-2015-0008.

[30] Qingdian L, Ying L, Jianping L. Yield and nutritional value of *Rosa laxa* Retz pollen. Scientia Hort, 1997;71(1–2):43–48. 10.1016/S0304-4238(97)00066-6.

[31] Saeheng S, Wongnawa M, Purintavaragul C. Chemical constituents and antioxidant activity of *Borussus flabellifer*, *Elaeis guineensis*, *Mimosa diplotricha* and *Mimosa pigra*. J Med Chem Drug Discov. 2012;3(1):52–57.

[32] Basuny AM, Arafat SM, Soliman HM (2013). Chemical analysis of olive and palm pollen: Antioxidant and antimicrobial activation properties. Wudpecker J Food Technol. 2013;1:14–21.

[33] Carpes ST, Mourao GB, De Alencar SM, Masson ML. Chemical composition and free radical scavenging activity of *Apis mellifera* bee pollen from Southern Brazil. Braz J Food Technol. 2009;12:220–229. 10.4260/BJFT2009800900016.

[34] Rebelo KS, Ferreira AG, Carvalho-Zilse GA. Physicochemical characteristics of pollen collected by Amazonian stingless bees. Ciência Rural. 2016;46(5):927–932. 10.1590/0103-8478cr20150999.

[35] Pascoal A, Rodrigues S, Teixeira A, Feás X, Estevinho LM. Biological activities of commercial bee pollens: antimicrobial, antimutagenic, antioxidant and anti-inflammatory. Food and Chemical Toxicology 2014;63:233–239. 10.1016/j.fct.2013.11.010 24262487

[36] Campos MGR, Bogdanov S, Almeida-Muradian LB, Szczesna T, Mancebo Y, Frigerio C. et al. Review article: Pollen composition and standardization of analytical methods. Journal of Apicultural Research 2008;47(2):156–163. 10.1080/00218839.2008.11101443.

[37] Arruda VAS, Pereira AAS, Freitas AS, Barth OM. Almeida-Muradian LB. Dried bee-pollen: B complex vitamins, physicochemical and botanical composition. Journal of Food Composition and Analysis 2013;29(2):100–105. 10.1016/j.jfca.2012.11.004.

[38] Delph LF, Johannsson MH, Stephenson AG. How environmental factors affect pollen performance: ecological and evolutionary perspectives. Ecology 1997;78(6):1632–1639. 10.1890/0012-9658(1997)078[1632:HEFAPP]2.0.CO;2.

[39] Pfahler PL, Linskens HF. Ash percentage and mineral content of maize (*Zea mays* L.) pollen and style. Theor and Appl Genet. 1974;45(1):32–36. 10.1007/BF00281171 24419219

[40] Zheng MY, Sun JJ, Wei YS, Zhang P, Geng W. Determination of Mineral Elements in Crocus Sativus L. by the Method of Microwave Digestion and ICP-OES. International Conference on Biomedical Engineering and Biotechnology; 2012 May 28–30; Macao, China: IEEE; 2012. p. 138–140. IEEE. 10.1109/iCBEB.2012.133.

[41] Kostic AZ, Pesic MB, Mosic MD, Dojcinovic BP, Natic MM, Trifkovic JD. Mineral content of bee pollen from Serbia. Arh Hig Rada Toksikol. 2015;66:251–258. 10.1515/aiht-2015-66-2630 26751856

[42] Harmanescu M, Popovici D, Gergen I. Mineral micronutrients composition of bee’s pollen. J. Agroaliment. Processes Technol. 2007;13(1):175–182.

[43] He FJ, MacGregor GA. Beneficial effects of potassium on human health. Physiol Plant. 2008;133(4):725–735. 10.1111/j.1399-3054.2007.01033.x 18724413

[44] Bello MO, Falade OS, Adewusi SR, Olawole NO. Studies on the chemical compositions and anti-nutrients of some lesser known Nigerian fruits. African J Biotechnol. 2008;7:3972–3979.

[45] Lee KH, Kim AJ, Choi EM. Antioxidant and anti-inflammatory activity of pine pollen extract in vitro. Phytotherapy Research: An International Journal Devoted to Pharmacological and Toxicological Evaluation of Natural Product Derivatives. 2009;23(1):41–48. 10.1002/ptr.2525 19107823

[46] Fatrcova-Sramkova K, Nozkova J, Kacaniova M, Mariassyova M, Rovna K, Stricik M. Antioxidant and antimicrobial properties of monofloral bee pollen. J Environ Sci Health. 2013;48:133–138. 10.1080/03601234.2013.727664 23305281

[47] Ceksteryte V, Kurtinaitiene B, Venskutonis PR, Pukalskas A, Kazernaviciute R, Balzekas J. Evaluation of antioxidant activity and flavonoid composition in differently preserved bee products. Czech J Food Sci. 2016;34:133–142. 10.17221/312/2015-CJFS.

[48] Zilic S, Vancetovic J, Jankovic M, Maksimovic V. Chemical composition, bioactive compounds, antioxidant capacity and stability of floral maize (*Zea mays* L.) pollen. J Func Food. 2014;10:65–74. 10.1016/j.jff.2014.05.007.

[49] Ku KM, Kim HS, Kim SK, Kang YH. Correlation analysis between antioxidant activity and phytochemicals in Korean colored corns using principal component analysis. J Agric Sci. 2014;6(4):1. 10.5539/jas.v6n4p1.

[50] Lundgren JG, Wiedenmann RN. Nutritional suitability of corn pollen for the predator *Coleomegilla maculata* (Coleoptera: Coccinellidae). J Insect Physiol. 2004;50(6):567–575. 10.1016/j.jinsphys.2004.04.003 15183287

[51] Marghitaş LA, Stanciu OG, Dezmirean DS, Bobiş O, Popescu O, Bogdanov S, et al. In vitro antioxidant capacity of honeybee-collected pollen of selected floral origin harvested from Romania. Food Chem. 2009;115(3):878–883. 10.1016/j.foodchem.2009.01.014.

[52] Chantarudee A, Phuwapraisirisan P, Kimura K, Okuyama M, Mori H, Kimura A, et al. Chemical constituents and free radical scavenging activity of corn pollen collected from *Apis mellifera* hives compared to floral corn pollen at Nan, Thailand. BMC Complem Altern M. 2012;12:45. 10.1186/1472-6882-12-45 22513008 PMC3488964

[53] Nicoli MC, Anese M, Parpine M. Influence of processing on the antioxidant properties of fruit and vegetables. Trends Food Sci Tech. 1999;10(3):94–100. 10.1016/S0924-2244(99)00023-0.

[54] Javanmardi J, Stushnoff C, Locke E, Vivanco JM. Antioxidant activity and total phenolic content of *Iranian Ocimum* accessions. Food Chem. 2003;83(4):547–550. 10.1016/S0308-8146(03)00151-1.

[55] Mascitelli L, Pezzetta F, Sullivan JL. The effect of polyphenols in olive oil on heart disease risk factors. Annals of Internal Med. 2007;146:394–394. 10.7326/0003-4819-146-5-200703060-00013 17339627

[56] Estevinho LM, Rodrigues S, Pereira AP, Feas X. Portuguese bee pollen: Palynological study nutritional and microbiological evaluation. Int J Food Sci Technol. 2012;47, 429–435. 10.1111/j.1365-2621.2011.02859.x.

[57] Fanali C, Dugo L, Rocco A. Nano-liquid chromatography in nutraceutical analysis: Determination of polyphenols in bee-pollen. Journal of Chromatography A 2013;1313: 270–274. 10.1016/j.chroma.2013.06.055 23880468

[58] Kostic AŽ, Trifunović BDS, Vukašinović IZ, Mačukanović-Jocić MP, Špirović Ivana Ž., Pešić MB, et al. Fatty acids of maize pollen–Quantification, nutritional and morphological evaluation. Journal of Cereal Science 2017;77:180–185. 10.1016/j.jcs.2017.08.004.

